# Editorial: The future of agricultural biosafety regulations

**DOI:** 10.3389/fbioe.2025.1735333

**Published:** 2025-11-10

**Authors:** Monica Garcia-Alonso, Clara Rubinstein, Stuart J. Smyth, Carmen Vicien

**Affiliations:** 1 Estel Consult Ltd., Berkshire, United Kingdom; 2 Institute for Scientific Cooperation in Environment and Health (ICCAS), Buenos Aires, Argentina; 3 University of Saskatchewan Saskatoon, Saskatoon, Canada; 4 School of Agronomy, University of Buenos Aires, Buenos Aires, Argentina

**Keywords:** biosafety, risk assessment, Ag biotech, adaptive regulations, gene editing, societal acceptance

## Introduction

Progressive and adaptive criteria for biosafety assessments require a collaborative effort across disciplines, experts and institutions around the globe to enable fit-for-purpose, risk appropriate regulations that promote the safe implementation of bio-innovations and their accessibility to society. A number of recent reports provide insights, recommendations, and actions in this direction (OECD, 2024; OECD, 2025; NESTA Report, 2019; Regulatory Horizons Council report, 2022). This is especially relevant, as food systems will have to sustain food security by producing greater yields per area, meet demands to be sustainable, and at the same time produce innovative, affordable foods.

Revisiting the scientific rationale for assessing gene technologies in the light of current knowledge and experience is critical for technologies to advance. Therefore, it was considered timely to compile a set of publications to provide an overview of the current challenges and opportunities for adapting biosafety considerations to support innovative biotechnological applications. This Research Topic features 12 publications authored by over 60 experts, representing realities and views from different world regions and sectors.

## Experiences and challenges in developing countries

Experts from Latin American and African countries share experiences and discuss regulatory frameworks, risk assessment approaches and innovative technologies, highlighting regulatory diplomacy and collaboration as enablers of functional and progressive frameworks.

In *“Experiences, learnings and perspectives in the regulation of agricultural biotechnology: the view from Argentina”*, Lewi et al. share their experiences with the revision process carried out between 2020 and 2023, based on the latest scientific knowledge. Collaborative initiatives and outreach actions implemented at the international level are also described, which resulted in the adoption of Argentina´s criteria for New Breeding Techniques in other geographies. This article stresses that continuous updating and training of risk assessors and inter-agency collaborations are key to enable adaptive frameworks and anticipate future challenges.

In “*Africa and zero hunger agenda: genome editing policy landscape, challenges and opportunities”*, Akinbo et al. comment on the historical opposing forces between the need to incorporate technology to ensure food security and strong positions against biotechnology as the main obstacle for adoption. They also review policies in five African countries regarding genome editing and continental initiatives that can promote agricultural innovations in the region.

Complementing this analysis, research conducted in African countries is presented by Rabuma et al., focused on the biosafety regulatory frameworks for genome editing and gene drive technologies. This study provides updated data that can be used to identify and address common challenges to develop effective regulatory frameworks going forward.

An additional complexity is addressed by Fernandez Rios et al., discussing the challenges and global trade implications of genome editing divergent regulatory criteria between regions. The article calls for more harmonized approaches that can not only facilitate trade but also stimulate the use of genome editing to address food security and mitigate climate change effects.

## There is a need for adaptive criteria and new models for regulatory oversight

Experts from different sectors offer their views on current regulatory models and propose advancements based on scientific knowledge and experience with risk assessment of gene technologies. Innovative applications in fruits and microorganisms are also discussed as cases that need adaptive criteria to advance.

In *“Naturally transgenic plants and the need to rethink regulatory triggers in biotechnology”*, Fernandez Rios et al. revisit the product vs. process regulatory approach for gene technologies, considering the complexity of plant genetics and the existence of naturally occurring transgenic plants, of which there is growing evidence. This article proposes to rethink the regulatory triggers for plant products derived from gene technologies, in particular, transgenesis.

Along these lines, two articles focus on GM microbial based biologicals. In the first, Karberg reviews the complexities of the microbial world, as the natural genetic exchange among microorganisms shows that natural transgenesis is common, making it difficult to label a microorganism as GM (or not). The article puts into question the scientific relevance of such labels for risk assessments and advocates for a more effective and science-based regulatory approach focused on the microorganisms’ actual functions.

In the second, Rubinstein et al. elaborate further on these arguments in *“Genetically modified microorganisms for agricultural use: an opportunity for the advancement of risk assessment criteria in Argentina”.* The article reflects a discussion conducted in Argentina, proposing a change in the current paradigm developed over 30 years ago for transgenic plants. The rationale is based on extensive scientific evidence and concludes that there is a need to adapt regulatory criteria to the microbial world.

Fruit improvement through gene technologies, is exemplified by Klocko in *“Apple improvement, traditional approaches, biotechnology options, and regulatory considerations*”. This work describes multiple apple breeding approaches with focus on transgenesis. As global biosafety regulations continue to develop and change, the article points to the need to develop guidelines for these cases, both for the cultivation and for import of engineered fruits, in this case, apple trees and apple derived products.

## On regulatory frameworks and the importance of perception

On the regulatory model side, Koch et al. offer the views of experienced developers of GM plants, pointing the 30 plus years of experience with risk assessment in many countries and calling to capitalize on this experience to avoid redundancy in country by country safety reviews of the same cases. The article proposes a global collaborative, harmonized model for risk assessment that is nimble and forward-looking, to keep up with the pace of innovations and enable broader access to their benefits.

Buchman and Kovak offer a US-focused analysis in *“A Call for Congressional Action: Revisiting the U.S. Coordinated Framework for the Regulation of Biotechnology”.* This Policy Brief examines the Coordinated Framework for the Regulation of Biotechnology developed almost 40 years ago and calls for substantial changes for current and future products. Recommendations ask, among others, for the creation of a centralized application submission portal, a horizon scanning for future products of biotechnology and streamlined regulations for familiar products.

On the other side of the Atlantic, Garcia Alonso et al. review the situation of GM crops in the EU, providing an historic framework and discussing the challenges posed by the current regulatory approach. The impacts of the rigid and outdated regulatory oversight of GMOs on innovation, EU farmers access to biotechnology technologies, trade implications and market concentration are discussed. Authors advocate for changes that can reduce the current complexity and unpredictability of the process, aligning the regulatory oversight of GM crops with current scientific knowledge and international practices.

Finally, in *“Agricultural biotechnology in the courts: judicial opinions and commentary”*, Kershen provides a thorough analysis of judicial opinions in different countries and their impacts on innovation and access to the benefits of biotechnology by society. The influence of public perception on decision makers and judicial decisions and the consequences for agricultural development are also discussed.

In summary, this Research Topic presents relevant perspectives and diverse international views on the necessary changes to support and advance innovation. These contributions provide scientific arguments emphasizing the importance of ensuring that agricultural biosafety regulations are fit for purpose, internationally harmonized - where possible- and forward looking, grounded on science and risk assessment. Regardless of geographical regions, this Research Topic shows the common challenges faced, the consensus on the need for a paradigm shift and the critical role of regulatory diplomacy in preparing for the future.
